# Lower-Limb Kinematic Reconstruction from Surface Electromyography Across Locomotor Tasks Using Shared Muscle Synergies

**DOI:** 10.3390/bioengineering13101112

**Published:** 2026-09-24

**Authors:** Bingyu Pan, Yexuan Wang, Mingzi Xiang

**Affiliations:** School of Sports Engineering, Beijing Sport University, Beijing 100084, China

**Keywords:** electromyography, muscle synergy, joint angle reconstruction, lower-limb kinematics

## Abstract

Surface electromyography (sEMG) reflects neuromuscular control, but multichannel recordings are high-dimensional and difficult to interpret. This study evaluated whether muscle-synergy activations provide a compact input representation for lower-limb kinematic reconstruction. Twelve-channel sEMG and hip, knee, and ankle angles were obtained from 120 healthy male participants performing seven tasks in the Gait120 dataset. A four-synergy representation was derived using nonnegative matrix factorization, and within-participant cross-task similarity was assessed. Under participant-wise five-fold cross-validation, fold-specific shared dictionaries were estimated exclusively from training participants, and separate task-specific models reconstructed joint trajectories across five locomotor tasks. ExtraTrees achieved the lowest RMSE in most task–joint combinations and was used for exploratory detailed comparisons. Shared-synergy activations reduced the regressor-input representation from 12 variables to four activation coefficients per time point. Task-level mean RMSEs were 5.60–6.29° for synergy activations and 5.52–6.28° for raw sEMG. After Holm correction, no statistically significant difference was detected in 14 of the 15 task–joint comparisons, while one favored synergy activations. These findings support shared-synergy activation as a compact and physiologically interpretable input representation and provide a basis for neuromuscularly informed modeling of lower-limb movement across locomotor conditions.

## 1. Introduction

Lower-limb joint kinematic estimation is an important topic in human movement analysis and has broad applications in rehabilitation training [1], intelligent prostheses [2], and wearable assistive-device control [3]. Accurate estimation of hip, knee, and ankle joint trajectories provides quantitative information about human locomotor function and enables continuous monitoring of movement performance beyond laboratory environments. Current approaches for obtaining lower-limb kinematics mainly include inertial measurement units (IMUs) [4,5], biomechanical modeling methods [6,7], and surface electromyography (sEMG) [8,9].

sEMG provides a non-invasive measurement of neuromuscular activation and has been widely used in biomedical signal processing applications, including movement intention recognition, human machine interaction, and rehabilitation assessment [10,11,12]. By capturing muscle activation patterns involved in movement generation, sEMG can offer predictive information before observable joint movement due to the temporal delay between muscle activation and mechanical response. Consequently, sEMG-based lower-limb kinematic reconstruction has attracted increasing attention for applications in wearable gait analysis, functional assessment, and real-time assistive-device control [13,14,15]. However, multichannel sEMG signals are high-dimensional, redundant, and susceptible to recording variability [16,17]. Furthermore, the relationship between muscle activation and joint motion is highly nonlinear and complex [18]. These challenges make it difficult to develop accurate, robust, and physiologically interpretable predictive models.

Previous studies have demonstrated the feasibility of sEMG-driven lower-limb kinematic estimation using both conventional machine learning and deep learning approaches. Coker et al. used knee muscle sEMG to train an artificial neural network for predicting future knee flexion angles during gait at prediction horizons of 50–200 ms, highlighting the potential of sEMG for anticipatory exoskeleton control [19]. Truong et al. showed that long short-term memory networks could estimate lower-limb joint angles and moments from sEMG signals across multiple daily activities. The findings indicated that sEMG alone contains useful information for reconstructing lower-limb biomechanics [8]. Zhao et al. further integrated lower-limb sEMG and inertial signals with random forest regression to predict hip and knee joint angles during walking, stair ascent, and uphill walking, demonstrating the value of wearable biosignals for multi-task joint-angle estimation [20]. More recently, data-driven models have increasingly adopted automatically extracted features or deep sequential architectures to improve the prediction of lower-limb gait time series from wearable sensor data [7]. Although these approaches have improved numerical prediction accuracy, most existing models rely on raw sEMG envelopes, handcrafted features, or black-box latent representations. Such representations can capture local muscle activation characteristics but do not explicitly encode the coordinated recruitment of multiple muscles. As a result, they provide limited insights into the underlying organization of neuromuscular control and may show reduced robustness when generalized across joints, subjects, and movement tasks.

Muscle synergy analysis provides a physiologically interpretable framework for studying the modular organization of neuromuscular control. According to muscle synergy theory, the central nervous system may simplify the control of multiple muscles by flexibly recruiting a limited number of muscle activation modules or synergies [21,22]. Using the non-negative matrix factorization (NMF) algorithm, multi-channel sEMG signals are decomposed into muscle weighting patterns and time-varying activation coefficients [23]. The muscle weighting matrix represents the relative contribution of each muscle to each synergy, whereas the activation coefficients describe the temporal recruitment of these synergies over the movement cycle. The seminal work by d’Avella et al. showed that combinations of a small number of time-varying muscle synergies could account for the muscle activation patterns underlying natural motor behaviors, supporting the idea that complex movements may be generated through the flexible recruitment of low-dimensional motor modules [21]. In human locomotion, Ivanenko et al. reported that five basic activation patterns could account for approximately 90% of the variance in multi-muscle EMG activity during walking, suggesting a modular structure underlying rhythmic lower-limb control [22]. Muscle synergy analysis has also been widely used in neurorehabilitation. Clark et al. found that post-stroke walking was characterized by a reduced number of motor modules and merging of healthy modules, and that these changes were associated with impaired locomotor performance [24]. Recent reviews further indicate that muscle synergies can be used to characterize motor impairment and rehabilitation-induced changes, although methodological variability and limited clinical translation remain important challenges [25].

These findings suggest that muscle synergy activation provides a compact and physiologically interpretable representation of task-relevant neuromuscular coordination. However, muscle synergy analysis has primarily been used to characterize differences in synergy number, muscle composition, and temporal activation across movement tasks, participant groups, and stages of rehabilitation [26,27]. Relatively few studies have examined whether synergy activation coefficients can serve as predictive low-dimensional inputs for reconstructing lower-limb joint kinematics. It therefore remains unclear whether muscle synergy activation preserves sufficient kinematic information to provide a compact, robust, and interpretable representation for reconstructing hip, knee, and ankle trajectories across multiple movement tasks.

In this study, we investigated whether muscle-synergy activation could serve as a low-dimensional neuromuscular representation for lower-limb kinematic reconstruction. Using a public dataset containing 12-channel sEMG and lower-limb joint angles across seven gait-related tasks, we first quantified within-participant cross-task similarity in task-specific synergy weights. Within participant-wise cross-validation, fold-specific shared dictionaries were estimated from training participants across five locomotor tasks, and task-specific models compared whole-cycle reconstruction using shared-synergy activation and raw sEMG in held-out participants. The shared-synergy representation reduced the input dimensionality supplied to the regressors from 12 to 4 channels, although all 12 sEMG channels remained necessary to calculate the activations. Using ExtraTrees for exploratory follow-up analyses, we further compared shared-synergy activation with four-component PCA features and activation derived from muscle-label-permuted dictionaries. These analyses provide methodological evidence for compact and interpretable sEMG representations across predefined locomotor contexts.

## 2. Materials and Methods

### 2.1. Dataset

This study used the publicly available Gait120 dataset, which contains synchronized full-body kinematics and surface electromyography (sEMG) recordings during common lower-limb locomotor and transitional movements [28]. The dataset was collected from 120 healthy adult male participants aged 20–59 years and includes seven tasks: level walking, stair ascent, stair descent, slope ascent, slope descent, sit-to-stand, and stand-to-sit. The complete dataset contains 6882 validated movement cycles across these tasks. The original data collection protocol, ethical approval, instrumentation, and processing procedures have been described in detail by Boo et al. [28].

In this study, we used the processed right lower-limb sEMG signals and the corresponding right lower-limb joint-angle trajectories. The sEMG data included 12 muscles: vastus lateralis (VL), rectus femoris (RF), vastus medialis (VM), tibialis anterior (TA), biceps femoris (BF), semitendinosus (ST), medial gastrocnemius (GM), lateral gastrocnemius (GL), medial soleus (SM), lateral soleus (SL), peroneus longus (PL), and peroneus brevis (PB). The target kinematic variables were the sagittal-plane right hip, knee, and ankle joint-angle trajectories.

### 2.2. Data Preprocessing

The present analysis used the processed sEMG and joint-angle data provided in the Gait120 dataset. As described by the dataset authors [28], the raw sEMG signals were mean-centered, band-pass filtered, full-wave rectified, corrected for outliers using a Hampel filter and smoothed using a root mean square envelope. For each participant, muscle, and task, the normalization reference was defined as the maximum amplitude observed across both the processed MVC recording and the corresponding task-specific sEMG signals. The task-specific sEMG signals were divided by this reference, segmented into movement cycles and interpolated to a uniform cycle length. Joint-angle trajectories were calculated using OpenSim (version 4.0) inverse kinematics and organized at the same movement-cycle level.

### 2.3. Muscle Synergy Extraction

Muscle synergies were extracted from the processed sEMG using NMF [29,30]. For each sEMG data matrix, NMF decomposes multi-channel muscle activity into a set of time-varying activation coefficients and muscle weighting patterns:

(1) X≈HW

where $X\in R_{+}^{T\times M}$ denotes the non-negative sEMG matrix, T is the number of time points, and M is the number of recorded muscles. The matrix $H\in R_{+}^{T\times K}$ contains the time-varying activation coefficients of the extracted synergies, and *K* is the number of synergies. $W\in R_{+}^{K\times M}$ contains the corresponding muscle weighting patterns.

NMF was implemented using scikit-learn with the coordinate-descent solver and the squared Frobenius reconstruction loss. No explicit regularization was applied to either factor matrix, and the $L_{1}$ mixing parameter was set to zero. NNDSVDa initialization and a convergence tolerance of $10^{-5}$ were used. The maximum number of iterations was 30,000 for participant–task decompositions used in the VAF analysis, 10,000 for those used in the cross-task synergy-similarity analysis, and 2500 for fold-specific shared-dictionary decompositions and fixed-dictionary projections. Fits that reached the applicable iteration limit and generated a ConvergenceWarning were classified as not converged; these fits were retained and flagged if their factor matrices were finite and nonnegative. A random state of 42 was specified for implementation consistency. Because NNDSVDa initialization was deterministic, no multiple-start solution selection was performed.

The number of muscle synergies was determined using variance accounted for (VAF). For each participant and task, NMF was repeated with the number of synergies ranging from K = 1 to K = 8. Reconstruction quality was quantified using both total VAF and muscle-specific VAF. Total VAF was calculated as [24,31]:

(2) $${VAF}_{total}=1-\frac{{\left\|X-HW\right\|}_{F}^{2}}{{\left\|X\right\|}_{F}^{2}}$$

where ${\left\|\cdot \right\|}_{F}$ denotes the Frobenius norm. Muscle-specific VAF was computed analogously for each sEMG channel by comparing the original and reconstructed trajectories of each muscle. For descriptive assessment, the minimum number of synergies satisfying ${VAF}_{total}\geq 0.9$ [32,33] and muscle-specific ${VAF}_{m}\geq 0.75$ [34,35] was identified for each participant and task.

### 2.4. Muscle Synergy Alignment and Cross-Task Similarity Analysis

For each participant and task, all available movement cycles were concatenated along the temporal dimension without cycle averaging, and NMF was performed separately. For subsequent synergy alignment, cross-task similarity analysis, and visualization, each row of the resulting muscle-weighting matrix W, representing one synergy across the 12 recorded muscles, was normalized to unit $L_{2}$ norm. The corresponding activation coefficients H were not used in these analyses and were therefore not rescaled. VAF was calculated from the original unnormalized NMF factors.

For each task, an initial task-level template containing K synergies was obtained by applying NMF to the temporally concatenated sEMG data from all available participants. Each participant’s normalized synergy vectors were matched to the current template using pairwise cosine similarity and the Hungarian assignment algorithm [36,37], with the assignment selected to maximize the total similarity across the *K* matched pairs. The aligned participant-level synergy vectors were then averaged elementwise, and each mean vector was normalized to unit $L_{2}$ norm to update the template. This matching, averaging, and template-updating procedure was repeated six times. The final template was defined as the task-average synergy matrix. When reporting relative muscle contributions, each synergy vector was additionally normalized to unit $L_{1}$ norm so that its 12 muscle weights summed to one.

Cross-task similarity was calculated separately at the participant level rather than from the task-average matrices. For each pair of tasks, the corresponding task-specific muscle-weighting matrices from every participant who completed both tasks were normalized row-wise and matched using the cosine-similarity-based Hungarian assignment procedure. The mean cosine similarity across the *K* matched synergy pairs was retained as that participant’s cross-task similarity score.

### 2.5. Prediction Models

To evaluate whether muscle synergy activations provide a compact and effective neuromuscular representation for lower-limb kinematic reconstruction, conventional machine-learning models and sequence-based deep-learning models were used to reconstruct hip, knee, and ankle joint-angle trajectories across multiple locomotor tasks. A separate regressor was trained for each task; data were not pooled across tasks, and no model parameters were shared between task-specific models.

The conventional machine learning models comprised ridge regression, partial least-squares regression, k-nearest-neighbor regression, and extremely randomized trees (ExtraTrees). Ridge regression provided a linear baseline with $L_{2}$ regularization [38], whereas partial least-squares regression modeled the relationships between high-dimensional predictors and multi-output joint trajectories through supervised latent components [39]. K-nearest-neighbor regression served as a non-parametric instance-based baseline [40]. ExtraTrees provided a nonlinear ensemble baseline for modeling complex relationships between inputs and outputs [41]. For these models, each input representation was vectorized over the full movement cycle, and the hip, knee, and ankle trajectories were predicted as a single flattened multi-output vector.

The sequence-based deep learning models comprised a temporal convolutional network (TCN) and a convolutional long short-term memory network (CNN-LSTM). The TCN used temporal convolutions to capture sequential dependencies across the movement cycle [42], whereas the CNN–LSTM combined convolutional feature extraction with recurrent modeling of longer-range temporal dependencies [43]. These models were included to test whether preserving the temporal organization of the input trajectories during training improved whole-cycle reconstruction. In contrast to the conventional regressors, the deep learning models received each input representation as a time-series sequence over the normalized movement cycle and predicted the corresponding multi-joint angle trajectories. Detailed prespecified configurations and training parameters for all prediction models are provided in Supplementary Table S1.

Within each outer fold, the 96 outer-training participants were further divided into 81 inner-training and 15 validation participants using a participant-wise GroupShuffleSplit with a configured validation fraction of 0.15. Random states of 43–47 were used for outer folds 1–5, respectively. The same inner split was used across tasks, input representations, and deep-learning architectures. The validation participants were used exclusively for early stopping and model-state selection.

After descriptive inspection of the outer-cross-validation performance of the six prespecified model families, ExtraTrees was selected post hoc for detailed representation comparisons and control analyses. These ExtraTrees-specific analyses were therefore treated as exploratory rather than confirmatory.

### 2.6. Shared-Dictionary Estimation and Cross-Validation

All prediction experiments were evaluated using participant-wise five-fold cross-validation. The outer folds were generated by applying GroupKFold with five splits, without shuffling, to samples ordered by participant identifier; therefore, no random seed was used for the outer split. Each fold contained 96 outer-training and 24 outer-test participants, and all cycles from each participant remained in the same partition. To obtain a unified muscle-synergy representation for joint-angle prediction across the five observed locomotor tasks and to assess generalization to unseen participants within each task, a fold-specific shared synergy dictionary, $W_{group}$, was constructed using only the training participants. All complete sEMG cycles from the five locomotor tasks were concatenated across training participants along the sample dimension without cycle averaging. Because no equalization or resampling was applied, participants and tasks contributed to shared-dictionary estimation in proportion to the number of available movement cycles they contributed. Each cycle contributed 101 temporal observations to the concatenated sEMG matrix. NMF was then applied to the concatenated matrix to estimate $W_{group}$.

For prediction, the fold-specific shared dictionary $W_{group}$ was retained at the original scale returned by NMF and held fixed. Each complete sEMG cycle from the training and held-out participants was projected separately onto this dictionary using the coordinate-descent transform implemented in scikit-learn’s NMF model. During this projection, only the nonnegative activation coefficients H were estimated, whereas $W_{group}$ remained unchanged. No post hoc rescaling of *H* was applied. The resulting 101×K activation matrix was specific to each participant, task, and movement cycle. During model fitting, each complete movement cycle was treated as one input–output sample. For conventional machine-learning models, the activation trajectories of all synergies over the normalized movement cycle were flattened into a single feature vector. For deep-learning models, the temporal sequence was retained, with synergy activations arranged as input channels over 101 normalized time points.

The held-out participants’ sEMG data were used only to calculate H from the fixed dictionary and generate predictions; they did not contribute to the estimation or updating of $W_{group}$ or the prediction-model parameters. Their joint-angle data were used only for final performance evaluation. Thus, dictionary estimation and model fitting were participant-disjoint, providing an assessment of generalization to unseen participants within each of the five observed locomotor tasks.

Model performance was quantified using root mean squared error (RMSE), the coefficient of determination ($R^{2}$), and Pearson correlation coefficient (r). Metrics were computed separately for each movement cycle and each predicted joint-angle trajectory across the normalized movement cycle. Cycle-level metrics were then averaged within each subject. Final performance was reported as the mean ± standard deviation across test subjects, thereby preventing participants with more movement cycles from disproportionately influencing the reported results.

### 2.7. Control Analyses

Several exploratory control analyses were conducted to evaluate whether the reconstruction results reflected muscle-synergy information rather than dimensionality reduction, movement-phase regularity, or the selected number of synergies. To ensure comparability, all prediction-based controls used the same task-specific model family and parameter configuration, together with identical fixed participant-wise five-fold cross-validation assignments. All data-dependent preprocessing transformations were estimated exclusively from the training participants within each outer fold and subsequently applied unchanged to the held-out participants.

First, a phase-only baseline was constructed to quantify the kinematic information available from the normalized movement phase without sEMG input. For each outer fold, task, and joint, trajectories were first averaged across cycles within each training participant. The 96 participant-specific mean trajectories were then averaged with equal participant weights to obtain the fold-specific 101-point baseline trajectory.

Second, a temporal circular-shift control was used to disrupt the phase correspondence between sEMG and joint kinematics while preserving the amplitude distribution, temporal structure, and intermuscular relationships of the sEMG signals. Each complete sEMG cycle was circularly shifted by an independently sampled integer offset of 20–81 samples, with the same offset applied to all 12 channels within that cycle. The analysis was repeated 10 times using deterministic task- and repeat-specific random seeds generated from the base seed 20260824. Activation coefficients were recalculated from the shifted sEMG using the fold-specific shared dictionary, and the task-specific regressors were retrained and evaluated using the shifted training and test inputs.

Third, four-component principal component analysis PCA-4 was used as a dimensionally matched linear control. Within each outer fold, a StandardScaler was fitted exclusively to the pooled sEMG observations from the five locomotor tasks of the training participants. PCA-4 was then fitted to the standardized training data. The fitted scaler and PCA transformation were applied unchanged to the training and held-out participants. The resulting PCA scores were supplied to the same task-specific regressors used for the four-dimensional synergy activation representation.

Fourth, a strict muscle-label permutation analysis was performed to examine whether the correspondence between muscle identity and the columns of the shared synergy dictionary contributed to reconstruction performance. For each outer fold, 200 randomly generated nonidentity permutations of the 12 muscle columns were evaluated. Within each permutation, the same column order was applied to all rows of the fold-specific shared dictionary, preserving its dimensionality, non-negativity, row norms, and distribution of synergy weights while disrupting their association with muscle labels. For each permuted dictionary, activation coefficients were re-estimated for both training and held-out participants, and the task-specific regressors were retrained before evaluation.

Finally, sensitivity to synergy number was examined by independently repeating the NMF decomposition, fold-specific shared-dictionary estimation, activation projection, and prediction procedures for K = 3, 4, 5, and 6. Within-participant cross-task synergy similarity was also recalculated at each K, with synergies matched between task pairs using cosine similarity and the Hungarian algorithm.

### 2.8. Statistical Analysis

The participant was the statistical unit. For all non-permutation comparisons, mean paired differences and unadjusted pointwise 95% confidence intervals were estimated using 10,000 participant-level percentile bootstrap resamples. Cohen’s $d_{z}$ was calculated as the mean paired difference divided by the sample standard deviation of the paired differences. For comparisons evaluated using the Wilcoxon signed-rank test, the test provided rank-based inference on whether the paired differences were centered at zero, whereas the mean paired differences, bootstrap confidence intervals, and Cohen’s $d_{z}$ values were reported as complementary estimation statistics describing the magnitude and uncertainty of the mean differences.

Raw sEMG and H were compared across the 15 task–joint combinations using two-sided Wilcoxon signed-rank tests. Differences were defined as raw-sEMG RMSE minus H RMSE, such that positive values favored H. *p*-values were Holm-adjusted across the 15 comparisons.

For the mean-trajectory and temporal circular-shift controls, the normality of the paired differences was assessed using the Shapiro–Wilk test. Two-sided paired *t*-tests were used when normality was not rejected (p≥0.05); otherwise, two-sided Wilcoxon signed-rank tests were applied. RMSE reductions were defined as the phase-only baseline RMSE minus the model RMSE for the mean-trajectory control and as the circularly shifted RMSE minus the temporally aligned RMSE for the temporal control. Positive values therefore indicated lower errors for the original temporally aligned model. Within each control analysis, Holm correction was applied across 40 comparisons comprising five tasks, four targets (hip, knee, ankle, and their mean), and two input representations.

PCA-4 and H were compared across the 15 task–joint combinations using two-sided Wilcoxon signed-rank tests. Paired differences were defined as PCA-4 RMSE minus H RMSE, such that positive values indicated lower errors for H, whereas negative values indicated lower errors for PCA-4. Mean paired differences, participant-level bootstrap 95% confidence intervals, and Cohen’s $d_{z}$ were reported as complementary estimation statistics. *p*-values were adjusted across the 15 comparisons using the Holm procedure.

For the strict muscle-label permutation analysis, held-out participant-level RMSEs were pooled across the five nonoverlapping outer test folds, with each participant contributing once. The corresponding fold-specific results for each permutation index were combined to form one complete permutation replicate. Task-level statistics were averaged across the hip, knee, and ankle, whereas joint-specific statistics were calculated separately. One-sided empirical *p*-values were determined from the proportion of permutations, yielding an RMSE no greater than that obtained with the original muscle-label organization, with 1 added to both the numerator and denominator. Holm correction was applied separately across the 5 task-level and 15 task–joint comparisons. The outer folds were treated as test partitions rather than independent replicates.

Statistical significance was set at Holm-adjusted *p* < 0.05. Analyses were conducted in Python 3.12.13 using scikit-learn 1.9.0 and PyTorch 2.2.2.

## 3. Results

### 3.1. Selection of the Number of Muscle Synergies

Participant- and task-specific NMF decompositions were performed for K = 1–8 to determine the number of muscle synergies required to reconstruct the sEMG signals. As shown in Figure 1a, the mean total VAF increased monotonically with K across all seven tasks. The largest improvements occurred between one and four synergies, after which the curves gradually approached a plateau. From K = 3 onward, the mean total VAF exceeded 0.90 for all tasks. For each participant and task, we then identified the minimum K at which the total VAF was at least 0.90 and the muscle-specific VAF was at least 0.75 for every recorded muscle (Figure 1b). For most tasks, the median required K ranged from three to four, although some participant–task decompositions required more than four synergies to satisfy both criteria.

At K = 4, all participants with available data satisfied the total VAF threshold in every task. The percentages for whom all 12 muscles also satisfied the muscle-specific VAF threshold were 83.3% for level walking, 62.5% for stair ascent, 55.0% for stair descent, 67.5% for slope ascent, 66.7% for slope descent, 88.0% for sit-to-stand, and 81.7% for stand-to-sit. Because the total VAF criterion was satisfied in all cases, these percentages also represent the proportions satisfying both criteria.

Considering the plateau in mean total VAF, the median participant-level requirements, and the need for a common dimensionality for comparisons across participants and tasks, K = 4 was adopted as the primary representation. This choice provided adequate overall sEMG reconstruction for all participants, although it did not satisfy the muscle-specific VAF criterion for every participant and task. Among the 6584 participant–task VAF decompositions performed for K = 1–8, 841 reached the 30,000-iteration limit and generated a convergence warning. Of these, 819 occurred at K = 1, whereas only 22 occurred across K = 3–8, including 6 of 823 decompositions at K = 4. All retained factor matrices were finite and nonnegative.

### 3.2. Task-Specific Muscle Synergy Structures and Cross-Task Similarity

At K = 4, all seven task-template decompositions converged. Participant-specific muscle synergies were extracted separately for each participant and task. After within-task alignment using cosine similarity and the Hungarian assignment algorithm, the aligned muscle-weighting vectors were averaged to obtain task-average synergy structures. For consistent presentation, the task-average synergies were reordered by matching them to the level walking reference (Figure 2).

The five locomotor tasks exhibited broadly similar synergy structures. Matched S1 was dominated by the plantar-flexor muscles, with additional contributions from the hamstrings in some tasks. Matched S2 was characterized by dominant contributions from the quadriceps muscles, particularly VL, RF, and VM, with variable contributions from the hamstrings across tasks. Matched S3 was mainly associated with the distal lower-leg muscles, particularly PL and PB, whereas matched S4 consistently exhibited the largest muscle weighting for TA across tasks. In contrast, sit-to-stand and stand-to-sit exhibited more task-specific weighting patterns. The quadriceps made prominent contributions to matched S2 and S3, although the relative weighting patterns differed between the two transitional tasks. Matched S1 showed more distributed contributions across thigh and lower-leg muscles, while the TA dominant structure of matched S4 was preserved.

Within-participant cross-task comparisons revealed a task-dependent pattern in muscle synergy similarity (Figure 3). For each participant and task pair, the four task-specific synergy vectors were matched one-to-one using the Hungarian algorithm, and the mean cosine similarity across the four matched synergies was used as the participant-level estimate. Across the 21 task pairs, the pair-specific mean similarities ranged from 0.621 to 0.907. For the 10 comparisons among the five locomotor tasks, all of which included 120 participants, mean similarities ranged from 0.775 to 0.907. The highest similarity was observed between stair ascent and slope ascent (mean ± SD: 0.907 ± 0.080; median [IQR]: 0.940 [0.845–0.972]; bootstrap 95% CI of the mean: 0.892–0.921), followed by level walking versus slope ascent (0.866 ± 0.086; 95% CI: 0.851–0.881) and level walking versus stair ascent (0.857 ± 0.090; 95% CI: 0.842–0.873). Comparisons between locomotor and transitional tasks generally showed lower and more variable participant-level similarities, with the lowest mean observed for stair descent versus sit-to-stand (0.621 ± 0.091). The sit-to-stand versus stand-to-sit comparison showed greater similarity than the locomotor–transition comparisons, although substantial inter-participant variability remained. Task pairs involving transitional movements included 107–115 participants because only participants with valid synergy estimates for both tasks were retained. Overall, the participant-level distributions indicate relatively greater similarity among locomotor tasks than between locomotor and transitional movements, while also demonstrating appreciable inter-participant variability in cross-task synergy structure.

### 3.3. Kinematic Reconstruction Using Shared Locomotor Synergy Activation

Six prediction models were evaluated using raw sEMG, shared-synergy activation H, and their concatenation. Separate task-specific regressors were trained using identical participant-wise cross-validation partitions for all input representations.

As shown in Figure 4, reconstruction performance varied across joints, tasks, and model families. ExtraTrees achieved the lowest RMSE in 13 of the 15 task–joint combinations. Based on this descriptive comparison, ExtraTrees was retained for exploratory participant-level comparisons of the input representations and subsequent control analyses. Results for all six model families and three input representations are presented in Figure 4. After averaging across the three joints, H yielded a numerically lower RMSE than raw sEMG in 24 of the 30 task–model combinations. When further averaged across tasks, its RMSE was lower for five of the six model families and nearly identical for ExtraTrees. Concatenating raw sEMG with H did not provide a consistent additional improvement. The corresponding joint-specific Pearson correlation coefficients are presented in Supplementary Figure S1.

In the exploratory ExtraTrees analysis, no statistically significant RMSE difference between raw sEMG and *H* was detected in 14 of the 15 task–joint combinations after Holm correction. The only significant difference occurred for the ankle during slope ascent, where H yielded a lower RMSE. The raw-sEMG minus H difference was 0.139° (unadjusted bootstrap 95% CI: 0.060–0.222°; Cohen’s $d_{z}$ = 0.309; Holm-adjusted *p* = 0.028). Across all 15 combinations, mean paired differences ranged from −0.182° to 0.164°, and the absolute Cohen’s $d_{z}$ values ranged from 0.022 to 0.309 (Supplementary Table S2). Thus, the observed differences were generally small and varied across tasks and joints, although formal equivalence was not established.

Within the offline whole-cycle ExtraTrees framework, H reduced the dimensionality of the representation supplied to the regressor from 101×12=1212 to 101×4=404 features per movement cycle, corresponding to a 66.7% reduction. This reduction concerned model-input dimensionality rather than the number of recorded signals, because all 12 sEMG channels remained necessary to calculate the four activation coefficients and an additional fixed-dictionary projection step was required.

### 3.4. Sensitivity to the Number of Muscle Synergies

Sensitivity analyses across K = 3–6 were conducted to assess the robustness of the cross-task similarity and kinematic reconstruction findings to the selected number of synergies. Participant- and task-specific NMF decompositions were repeated independently for each value of K. In the main K = 4 similarity analysis, 40 of 823 participant–task decompositions reached the 10,000-iteration limit and generated a convergence warning. In the independent K = 3–6 sensitivity analysis, the corresponding numbers were 11, 37, 72, and 75 of 823 at K = 3, 4, 5, and 6, respectively. These decompositions were retained and flagged, and the similarity results were computed using the resulting factors.

The cross-task similarity pattern remained stable across the examined values of K (Figure 5). Across the 10 pairs formed by the five locomotor tasks, the mean within-participant matched cosine similarities were 0.810, 0.824, 0.824, and 0.825 for K = 3, 4, 5, and 6, respectively. The corresponding ranges across task pairs were 0.748–0.922, 0.775–0.907, 0.795–0.899, and 0.794–0.883. Across all values of K, similarities among the locomotor tasks remained relatively high, similarities between locomotor and transitional tasks remained lower, and sit-to-stand and stand-to-sit remained relatively similar to each other.

In the exploratory ExtraTrees sensitivity analysis, reconstruction performance was broadly stable across K = 3–6 (Figure 6). All 20 fold-specific shared-dictionary decompositions across K = 3–6 converged. All 25 fixed-dictionary projections used in the K = 4 prediction analysis also converged. The mean RMSEs averaged across the 15 task–joint combinations were 5.982°, 5.947°, 5.897°, and 5.889° for K = 3, 4, 5, and 6, respectively. Relative to K = 4, the mean RMSE changed by +0.035° at K = 3, −0.050° at K = 5, and −0.058° at K = 6. Across individual task–joint combinations, the range between the highest and lowest RMSEs over K = 3–6 was 0.018–0.264°. Although K = 5 and K = 6 yielded slightly lower RMSEs on average, the magnitude of these changes was small.

Overall, the cross-task similarity structure and kinematic reconstruction performance were broadly stable across K = 3–6. These findings indicate that the main results were not specific to K = 4, while also showing a modest trade-off between representational compactness and prediction accuracy at larger values of K.

### 3.5. Control Analyses of Input Representation and Temporal Information

Based on its descriptive performance across the six prespecified model families, ExtraTrees was used for exploratory follow-up analyses. These analyses evaluated whether reconstruction performance was associated with dimensionality reduction, muscle-label organization, task- and phase-dependent regularity, or temporal alignment.

#### 3.5.1. Four-Dimensional PCA Control

PCA-4 yielded task-level mean RMSEs of 5.55–6.30°, compared with 5.60–6.29° for the shared-synergy activation coefficients H. The corresponding task-level $R^{2}$ values were 0.583–0.829 for PCA-4 and 0.568–0.829 for H, while Pearson’s r values were 0.946–0.971 and 0.946–0.971, respectively. Across the 15 task–joint combinations, paired differences defined as PCA-4 RMSE minus H RMSE ranged from −0.256° to 0.114°. Seven differences numerically favored the shared-synergy representation, whereas eight favored PCA-4. The absolute Cohen’s $d_{z}$ values ranged from <0.001 to 0.219, and pointwise bootstrap 95% confidence intervals included zero in 12 of the 15 comparisons. Although three pointwise confidence intervals excluded zero, none of the corresponding comparisons remained statistically significant after Holm correction across the 15 task–joint combinations (adjusted *p* = 0.061–1.000, Supplementary Table S3). Thus, no statistically detectable RMSE advantage specific to H was identified. Nevertheless, H retained a nonnegative muscle-synergy structure and provided components that were more directly interpretable in terms of coordinated muscle recruitment than the variance-based PCA features.

#### 3.5.2. Muscle-Label Permutation Control

To determine whether reconstruction performance depended on the original correspondence between muscle labels and synergy weights, a strict muscle-label permutation control was performed using 200 nonidentity permutations within each outer fold. The original dictionary yielded a lower three-joint mean RMSE than the permutation distribution for stair ascent (5.787° versus 5.907 ± 0.041°; Holm-adjusted empirical *p* = 0.0249) and slope ascent (5.771° versus 5.909 ± 0.037°; Holm-adjusted empirical *p* = 0.0249). No statistically significant advantage was detected for level walking, stair descent, or slope descent. Complete task-level results are provided in Supplementary Table S4. At the joint-specific level, none of the 15 comparisons remained significant after Holm correction (minimum adjusted *p* = 0.0746). These results provide evidence that the original muscle-label organization contributed to reconstruction performance in selected ascent tasks, but not consistently across all locomotor conditions.

#### 3.5.3. Phase-Only Mean-Trajectory Control

To determine whether raw sEMG and H improved reconstruction beyond task- and phase-dependent trajectory regularity, their ExtraTrees predictions were compared with fold-specific mean joint-angle trajectories calculated exclusively from the outer-training participants. For the three-joint mean RMSE, the reductions relative to the phase-only baseline ranged from 0.585° to 1.126° (8.5–16.3%) across the four stair and slope tasks and were statistically significant for both input representations after Holm correction (all adjusted *p* < 0.001). During level walking, the reductions were smaller and nonsignificant for both raw sEMG (0.150°, 95% CI: −0.057° to 0.348°; adjusted *p* = 0.901) and H (0.062°, 95% CI: −0.181° to 0.293°; adjusted *p* = 0.988). Complete task- and joint-specific results are provided in Supplementary Table S5.

#### 3.5.4. Temporal Circular-Shift Control

To determine whether reconstruction performance depended on temporal correspondence between the sEMG-derived inputs and joint-angle trajectories, ExtraTrees models using temporally aligned inputs were compared with models trained and evaluated using circularly shifted inputs. For the three-joint mean RMSE, the aligned inputs reduced error by 0.328–0.828° (5.0–12.5%) across the four stair and slope tasks, with all differences remaining statistically significant after Holm correction (all adjusted *p* < 0.001). During level walking, the aligned raw sEMG reduced the RMSE by 0.110° (95% CI: 0.016° to 0.203°; adjusted *p* = 0.004), whereas the reduction for aligned H was smaller and nonsignificant (0.030°, 95% CI: −0.093° to 0.151°; adjusted *p* = 0.658). Complete task- and joint-specific results are provided in Supplementary Table S6.

## 4. Discussion

This study examined whether shared muscle-synergy activation could provide a compact and interpretable representation for lower-limb kinematic reconstruction across five predefined locomotor tasks. Participant-level analyses showed relatively high similarity among the muscle-synergy structures of the locomotor tasks. ExtraTrees achieved the lowest RMSE in 13 of the 15 task–joint combinations. In the exploratory ExtraTrees analysis, H produced generally small RMSE differences relative to raw sEMG: 14 comparisons were not statistically significant after Holm correction, while 1 favored H. The main advantages of H therefore lie in compactness and interpretability rather than superior prediction accuracy.

The greater similarity among locomotor tasks than between locomotor and transitional tasks is consistent with the muscle-synergy framework, in which related movements recruit partially shared muscle-weighting modules [31,34]. The distinct biomechanical requirements of sit-to-stand and stand-to-sit may explain their more task-specific structures [44]. Sensitivity analyses across K = 3 to K = 6 showed broadly stable similarity and prediction patterns, supporting K = 4 as a practical common representation. At K = 4, all participant–task decompositions met the total VAF criterion, while the proportion satisfying all muscle-specific VAF thresholds varied across tasks, reflecting participant- and task-dependent differences in sEMG complexity.

The strong performance of ExtraTrees may reflect the fixed-length, phase-aligned structure of the present prediction problem, which allowed nonlinear muscle–time-point interactions to be modeled directly. Under these conditions, the additional temporal modeling capacity of TCN and CNN–LSTM may have provided limited benefit. Similar findings have been reported for tree-based models in sEMG-based joint-angle prediction [45]. The present comparison reflects the performance of the prespecified model configurations under a common participant-wise cross-validation framework. Moreover, the 66.7% reduction concerned only the dimensionality supplied to the regressor. All 12 sEMG channels remained necessary to calculate H, and an additional projection step was introduced; computational efficiency and reduced sensor requirements were not demonstrated.

PCA-4 produced reconstruction errors similar to those obtained using H, indicating that the observed predictive performance was not unique to the synergy representation. However, H retained a nonnegative muscle-weighting structure that was more directly interpretable in terms of coordinated muscle recruitment. The original muscle-label organization outperformed the permutation distribution only for the three-joint mean RMSEs of stair and slope ascent, with no significant joint-specific comparisons. Its contribution therefore appeared task-dependent rather than universal. The phase-only and circular-shift controls further showed that raw sEMG and H provided temporally relevant information beyond the mean joint-angle trajectory in the stair and slope tasks, whereas their additional contribution during level walking was smaller.

Several limitations should be acknowledged. The Gait120 dataset included only healthy male participants, requiring external validation in female and clinical populations. Participant-wise cross-validation assessed generalization to unseen participants within predefined tasks, but not to unseen movement tasks, because all five tasks contributed to the shared dictionary and separate regressors were trained for each task. ExtraTrees was retained for detailed analysis after inspection of the same outer cross-validation results subsequently interpreted; therefore, the ExtraTrees-specific representation comparisons and control analyses should be considered exploratory. The within-analysis Holm corrections did not account for this model-family selection. Amplitude normalization incorporated task-specific sEMG from each test participant, while cycle normalization required access to complete movement cycles. Accordingly, the present evaluation should be interpreted as offline whole-cycle reconstruction rather than fully prospective real-time prediction. Future studies should examine leave-one-task-out transfer, normalization based exclusively on calibration data available before testing, and streaming sEMG windows for predicting future joint angles.

## 5. Conclusions

This study evaluated activation coefficients derived from a shared locomotor synergy dictionary as a four-dimensional input representation for whole-cycle lower-limb kinematic reconstruction across five predefined locomotor tasks. Participant-level analyses revealed relatively high cross-task similarity among the locomotor muscle-weighting structures. In the exploratory ExtraTrees comparison, paired RMSE differences between H and raw sEMG were generally small: No statistically significant difference was detected in 14 of the 15 task–joint comparisons, while 1 favored H. The activation representation reduced the dimensionality supplied to the prediction model by 66.7% and provided direct interpretation in terms of coordinated muscle recruitment, although all 12 sEMG channels remained necessary to calculate the activations. These findings support shared-synergy activation as a compact and interpretable representation for participant-independent kinematic reconstruction across the locomotor conditions examined.

## Figures and Tables

**Figure 1:**
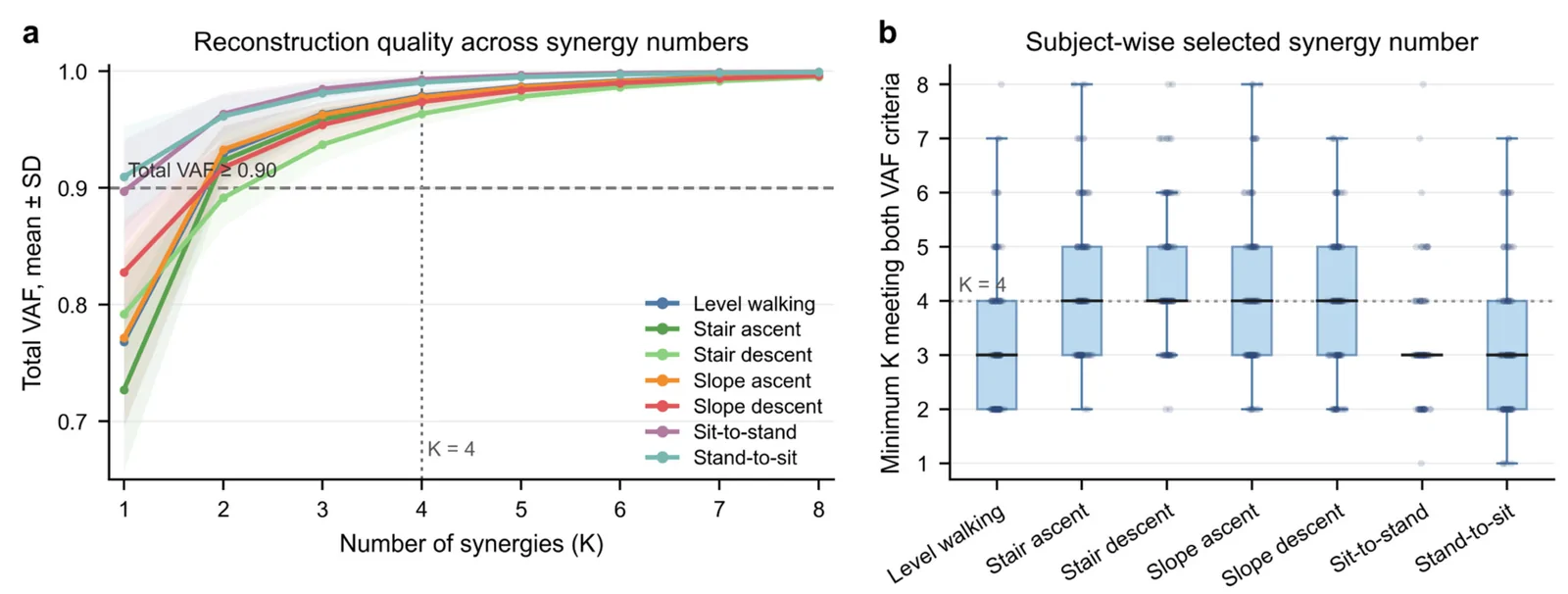
Variance-accounted-for analysis for selection of the number of muscle synergies. (**a**) Subject-wise total VAF across K = 1–8 for each task. Solid lines indicate the mean, and shaded bands represent ±1 standard deviation across participants. (**b**) Distribution of the minimum K satisfying a total VAF of ≥0.90 and a muscle-specific VAF of ≥0.75 across participants and tasks.

**Figure 2:**
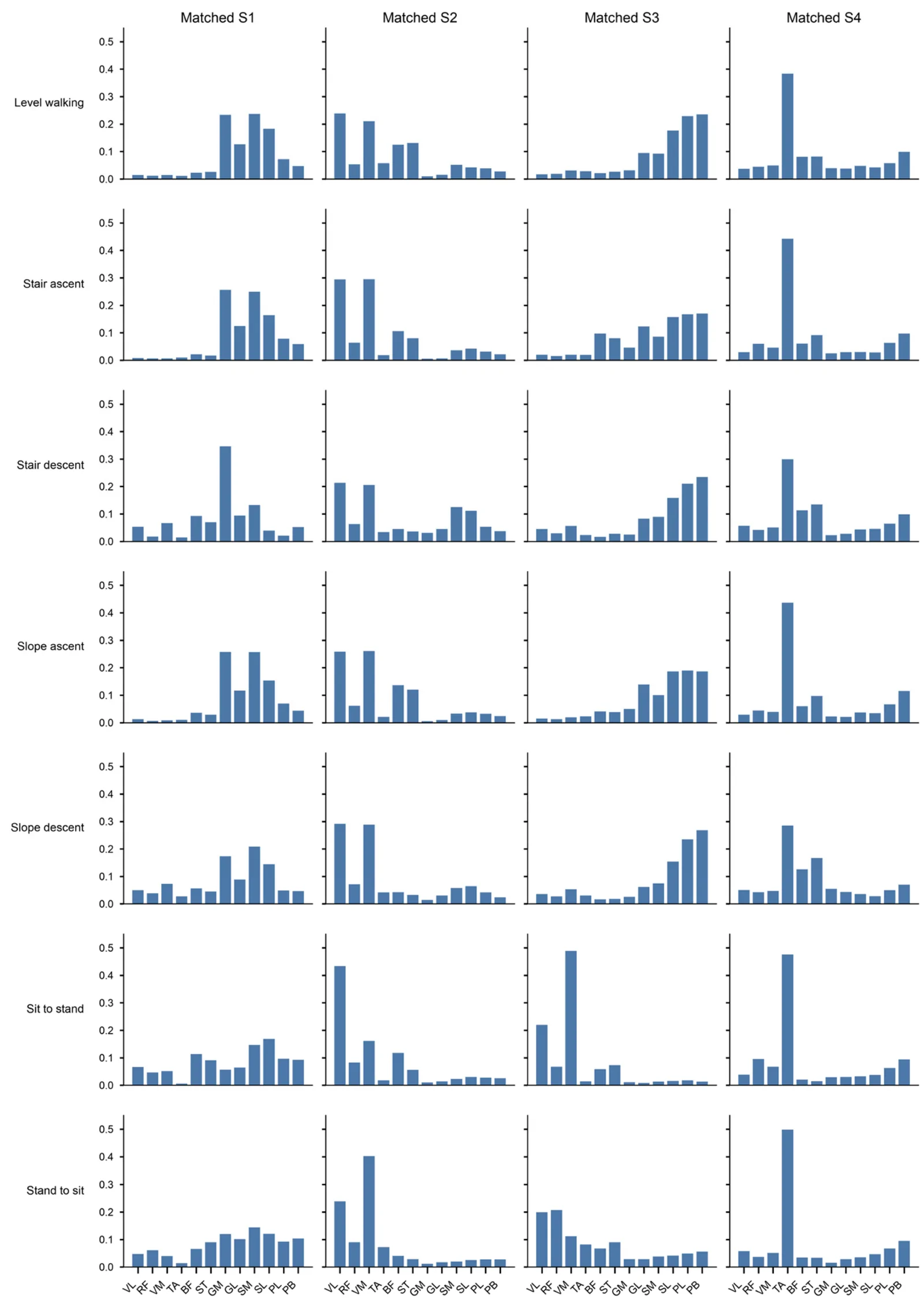
Task-average muscle-weighting patterns for K = 4 across the seven movement tasks. Rows represent tasks, and columns represent the four matched synergies (S1–S4). Within each task, participant-specific muscle-weighting vectors were aligned using cosine similarity and the Hungarian algorithm and then averaged. Task-average synergies were reordered relative to level walking. Bars show the relative weights of the 12 muscles.

**Figure 3:**
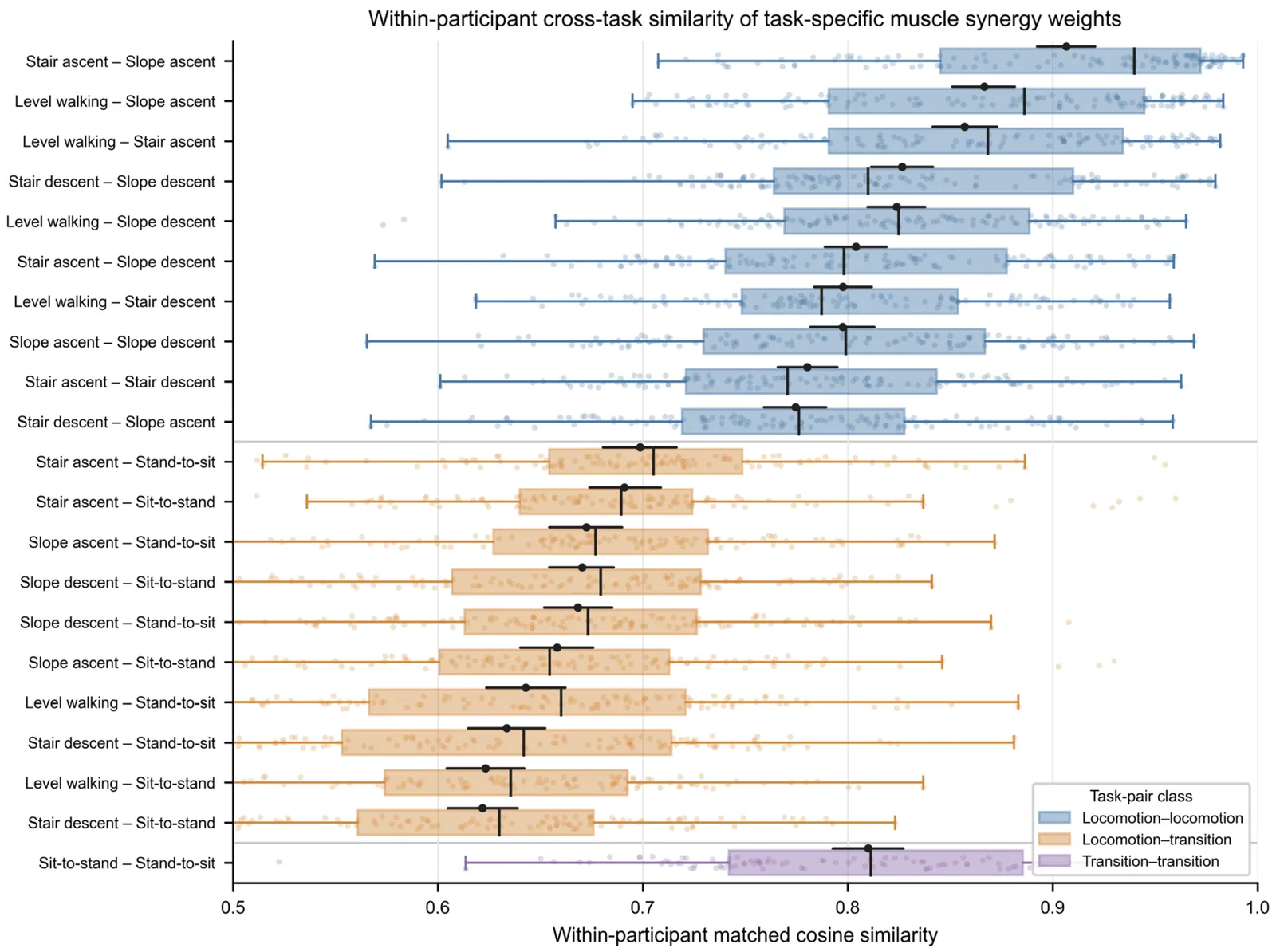
Within-participant cross-task similarity of task-specific muscle synergy weights. Boxes show the median and interquartile range, whiskers represent 1.5 times the interquartile range, and light-colored dots represent participants. Black dots and lines indicate the mean and bootstrap 95% confidence interval. Colors distinguish the three task-pair classes.

**Figure 4:**
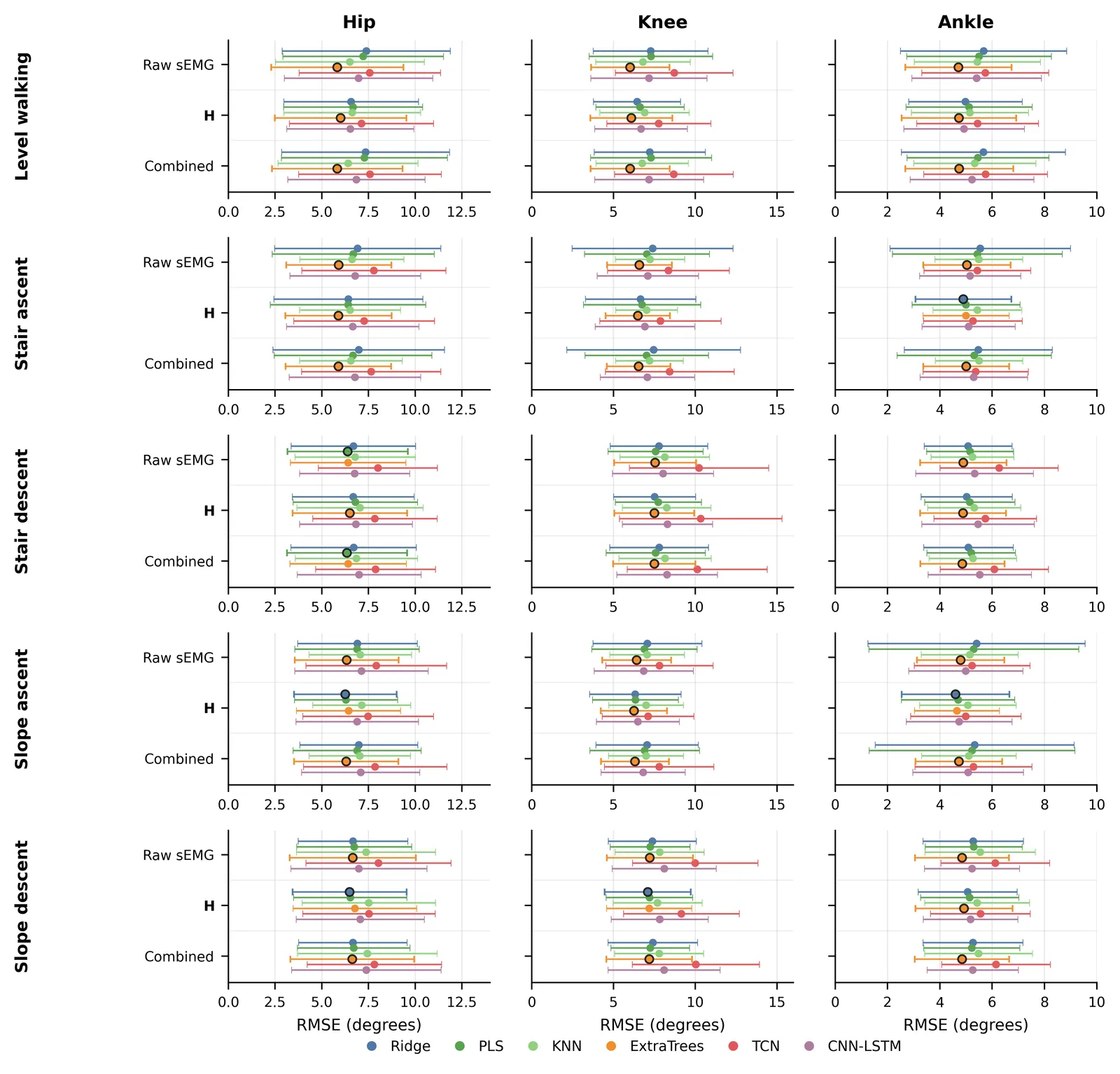
Joint-angle reconstruction RMSEs across five locomotor tasks. Columns represent the hip, knee, and ankle, and rows represent the five tasks. Points and error bars show the participant-level mean ± SD for six models using raw sEMG, H, or their combination. Black-edged circles indicate the model with the lowest mean RMSE within each input representation for each task–joint combination. Lower values indicate better performance. Each task-specific estimate included 120 participants.

**Figure 5:**
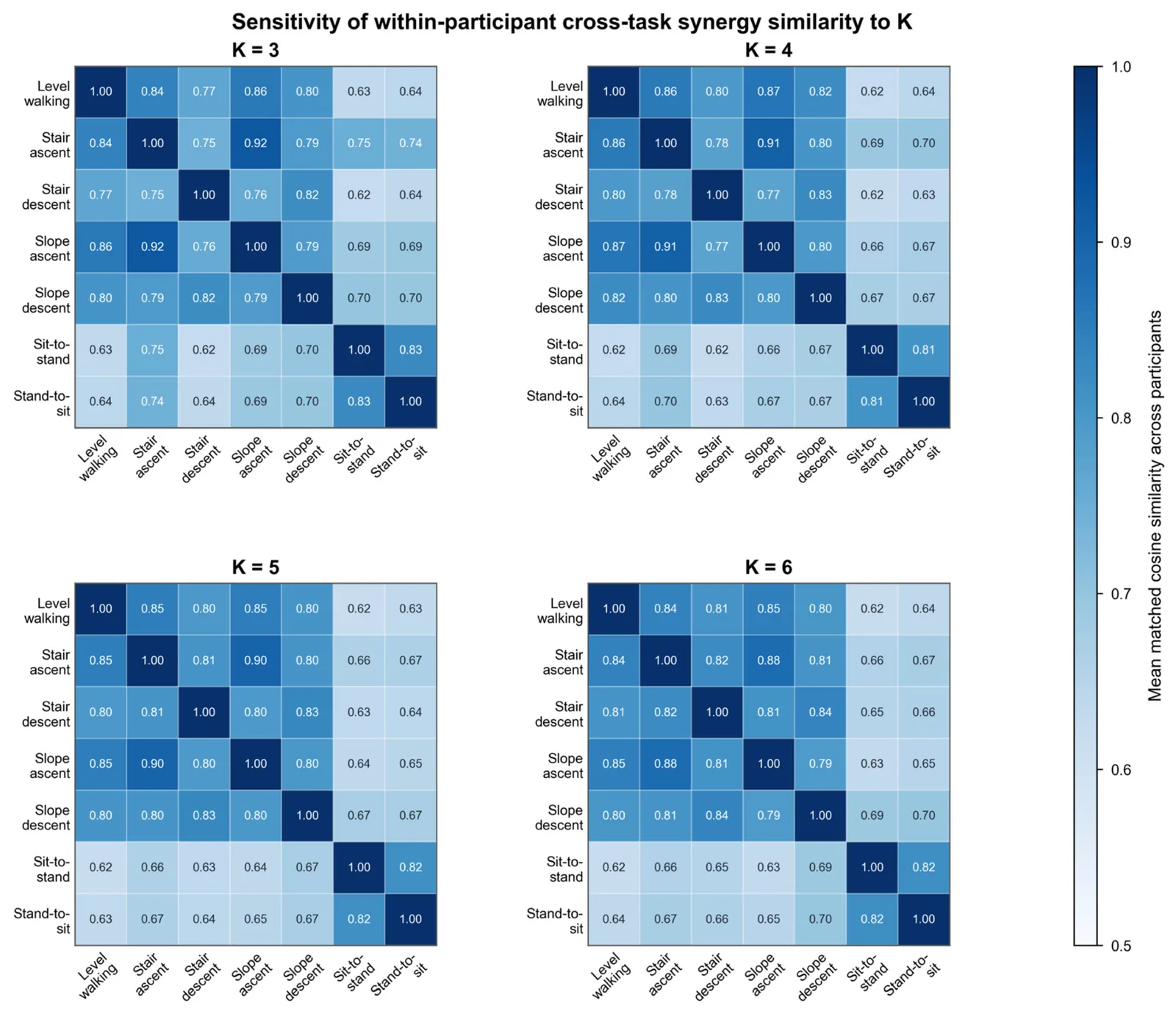
Sensitivity of within-participant cross-task synergy similarity to K. Cells show mean cosine similarity across participants after Hungarian matching. NMF was repeated independently for *K* = 3–6.

**Figure 6:**
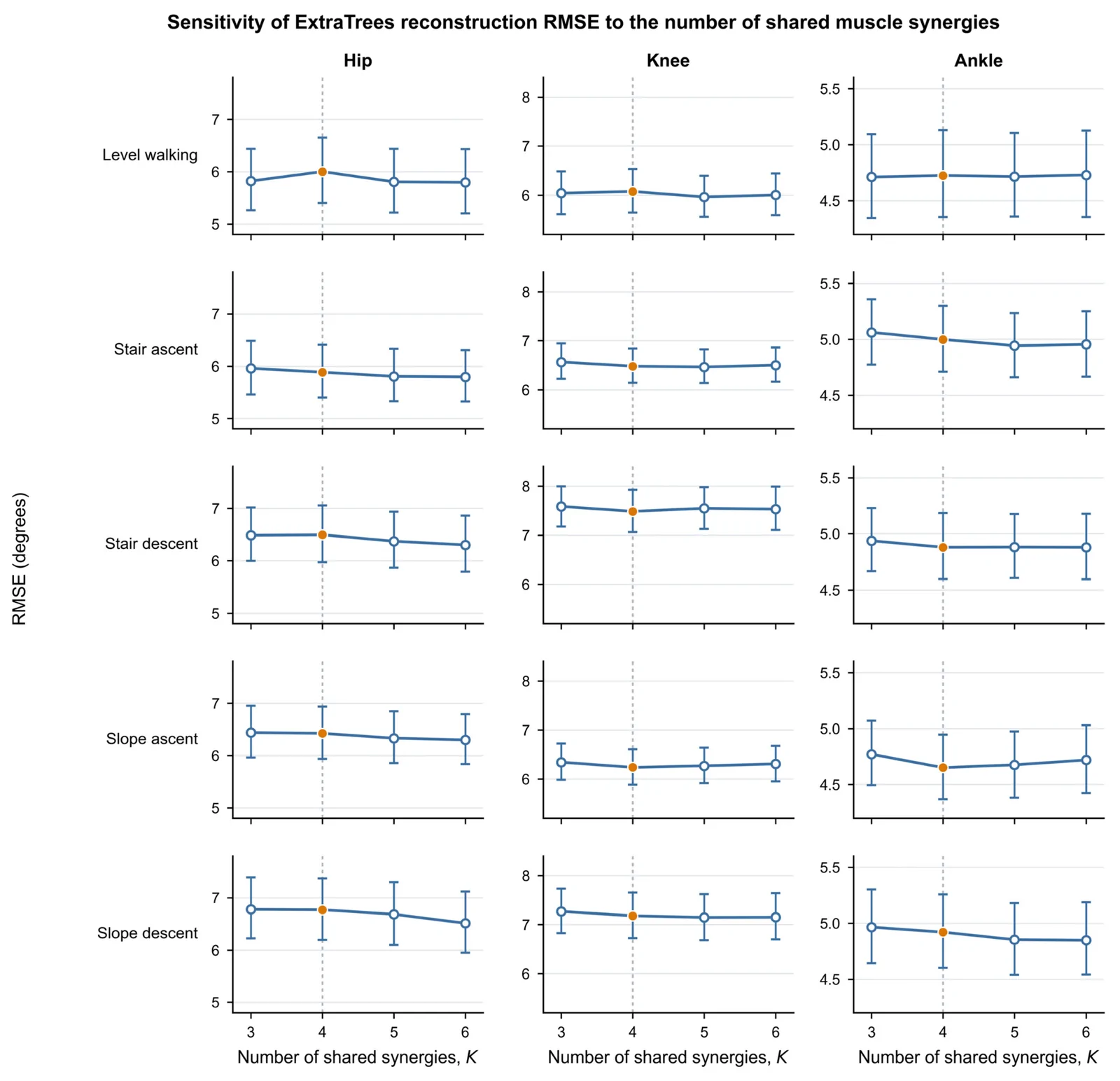
Sensitivity of ExtraTrees reconstruction RMSE to the synergy number. Rows represent tasks and columns represent joints. Points show participant means, and error bars show bootstrap 95% confidence intervals (n = 120). Orange markers indicate K = 4.

## Data Availability

The data presented in this study are openly available in Figshare at https://doi.org/10.6084/m9.figshare.27677016.v1, reference number 27677016.

## Supplementary Materials

The following supporting information can be downloaded at: https://www.mdpi.com/article/10.3390/bioengineering13101112/s1, Table S1: Prespecified model configurations of the prediction models; Figure S1: Pearson correlations between reconstructed and reference joint-angle trajectories; Table S2: Participant-level paired comparisons of reconstruction RMSEs between raw sEMG and H; Table S3: Participant-level paired comparison of reconstruction RMSEs between PCA-4 and shared-synergy activation using ExtraTrees; Table S4: Task-level results of the strict muscle-label permutation analysis; Table S5: Comparison of ExtraTrees reconstruction performance with the fold-specific phase-only mean-trajectory baseline; Table S6: Effect of temporal circular shifting on ExtraTrees reconstruction performance.

## Abbreviations

The following abbreviations are used in this manuscript:

CNN–LSTM	convolutional neural network–long short-term memory
EMG	electromyography
NMF	non-negative matrix factorization
RMSE	root mean square error
sEMG	surface electromyography
TCN	temporal convolutional network
VAF	variance accounted for
