# Supplementary material for: Lower-Limb Kinematic Reconstruction from Surface Electromyography Across Locomotor Tasks Using Shared Muscle Synergies
Source: Bioengineering (Basel). 2026 Sep 24;13(10):1112. doi: 10.3390/bioengineering13101112 (PMC13701826; doi:10.3390/bioengineering13101112)
Supplement: Supplementary file 1 [file bioengineering-13-01112-s001.zip › bioengineering-4537900-supplementary.pdf]

Supplementary Table S1. Prespecified configurations and training settings for the prediction models.

Model or procedure	Configuration
Feature scaling	Ridge, PLS, and KNN inputs were standardized using outer-training-fitted scalers; PLS additionally used internal scaling. ExtraTrees required no scaling. Deep-learning inputs and targets were standardized using inner-training data only. All fitted scalers were applied unchanged to validation and test data.
Ridge	α=100; solver = "auto"; intercept fitted; tolerance =10^{-4}.
PLS	24 components; internal scaling enabled; maximum iterations = 500; tolerance = 10^{-6}
KNN	Eight neighbors; distance weighting; Euclidean distance (Minkowski metric with $p = 2$); algorithm = "auto", leaf size = 30.
ExtraTrees	120 trees; squared-error criterion; maximum depth = 18; maximum features = 0.35; minimum leaf size = 2; minimum split size = 2; no bootstrap; random seed = 42.
CNN–LSTM	Two Conv1D layers with 32 filters and kernel sizes of 5 and 3; ReLU after each convolution; dropout =0.10 after the first convolution; one-layer bidirectional LSTM with 48 hidden units per direction; time-distributed linear output layer with three outputs per time point.
TCN	Three residual blocks with 48 filters per block; two Conv1D layers per block; kernel size =3; dilations =1,2 and 4; ReLU and dropout =0.12 after each convolution; final 1×1 convolution with three output channels.
Deep-learning training	AdamW optimizer learning rate = 1×10^{-3}; weight decay = 1×10^{-4}; batch size = 32; MSE loss; maximum epochs = 80; gradient clipping with a maximum norm of 1.0. Early stopping used an outer-training participant subset with patience = 10 and minimum improvement = 1×10^{-5}; the best model state was restored. Seed = 42 + outer-fold number.

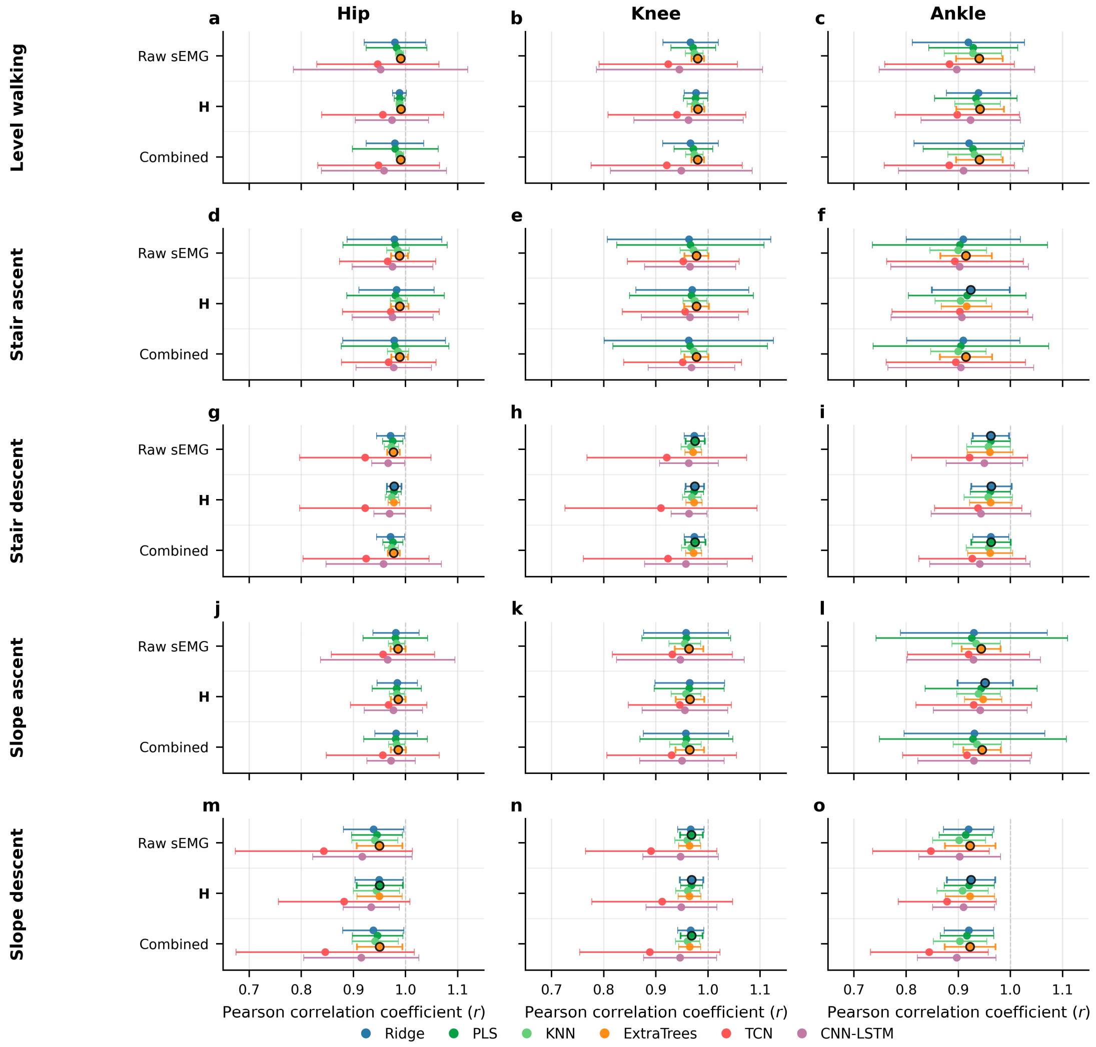

Supplementary Figure S1. Pearson correlations between reconstructed and reference joint-angle trajectories. Panels show the hip, knee, and ankle across five locomotor tasks and three input representations. Points and error bars indicate the participant-level mean ± SD. Each task-specific estimate included 120 participants.

Supplementary Table S2. Participant-level paired comparisons of reconstruction RMSE between raw sEMG and H

Locomotor task	Joint	Raw sEMG $-H°$ (95% CI)	Cohen’s d_z	Holm-adjusted P
Level walking	Hip	−0.182 (−0.442, 0.067)	−0.128	1
Level walking	Knee	−0.065 (−0.213, 0.082)	−0.078	1
Level walking	Ankle	−0.017 (−0.151, 0.112)	−0.022	1
Stair ascent	Hip	0.016 (−0.094, 0.129)	0.025	1
Stair ascent	Knee	0.099 (0.032, 0.171)	0.255	0.211
Stair ascent	Ankle	0.032 (−0.023, 0.089)	0.103	1
Stair descent	Hip	−0.093 (−0.264, 0.082)	−0.096	1
Stair descent	Knee	0.048 (−0.103, 0.201)	0.057	1
Stair descent	Ankle	0.012 (−0.079, 0.106)	0.023	1
Slope ascent	Hip	−0.103 (−0.235, 0.021)	−0.143	1
Slope ascent	Knee	0.164 (0.064, 0.271)	0.285	0.266
Slope ascent	Ankle	0.139 (0.060, 0.222)	0.309	0.028
Slope descent	Hip	−0.120 (−0.300, 0.057)	−0.119	1
Slope descent	Knee	0.042 (−0.107, 0.190)	0.049	1
Slope descent	Ankle	−0.077 (−0.163, 0.008)	−0.160	1

Supplementary Table S3. Participant-level paired comparisons of reconstruction RMSE between PCA-4 and shared-synergy activation using ExtraTrees.

Locomotor task	Joint	PCA-4 − H, ° (95% CI)	d_z	Holm-adjusted (P)
Level walking	Hip	−0.256 (−0.509 to −0.013)	−0.182	1
Level walking	Knee	−0.000 (−0.172 to 0.169)	−0.000	1
Level walking	Ankle	0.091 (−0.022 to 0.202)	0.145	0.904
Stair ascent	Hip	−0.032 (−0.158 to 0.096)	−0.045	1
Stair ascent	Knee	0.030 (−0.072 to 0.137)	0.052	1
Stair ascent	Ankle	0.012 (−0.057 to 0.084)	0.031	1
Stair descent	Hip	−0.046 (−0.200 to 0.109)	−0.053	1
Stair descent	Knee	0.114 (−0.047 to 0.268)	0.129	1
Stair descent	Ankle	−0.018 (−0.102 to 0.066)	−0.039	1
Slope ascent	Hip	−0.100 (−0.233 to 0.035)	−0.132	1
Slope ascent	Knee	0.086 (−0.018 to 0.195)	0.146	1
Slope ascent	Ankle	0.104 (0.010 to 0.202)	0.195	0.846
Slope descent	Hip	−0.205 (−0.375 to −0.043)	−0.219	0.061
Slope descent	Knee	0.012 (−0.163 to 0.190)	0.011	1
Slope descent	Ankle	−0.034 (−0.149 to 0.076)	−0.055	1

Supplementary Table S4. Task-level results of the strict muscle-label permutation analysis.

Task	Original RMSE °	Permuted RMSE, mean ± SD °	Holm-adjusted P
Level walking	5.603	5.538 ± 0.061	0.8806 (0.8806)
Stair ascent	5.788	5.907 ± 0.041	0.0050 (0.0249)
Stair descent	6.288	6.381 ± 0.048	0.0398 (0.1194)
Slope ascent	5.773	5.909 ± 0.037	0.0050 (0.0249)
Slope descent	6.289	6.367 ± 0.059	0.1045 (0.2090)

Table S5. Comparison of ExtraTrees reconstruction performance with the fold-specific phase-only mean-trajectory baseline

Task	Target	Input	Original/control RMSE (°)	RMSE reduction (95% CI), °	Holm-adjusted P
Level walking	Hip	Raw sEMG	5.824/5.947	0.123 (−0.173, 0.423)	1.000
Level walking	Hip	H	6.006/5.947	−0.059 (−0.440, 0.308)	1.000
Level walking	Knee	Raw sEMG	6.015/6.243	0.228 (−0.025, 0.483)	0.751
Level walking	Knee	H	6.080/6.243	0.164 (−0.131, 0.446)	0.901
Level walking	Ankle	Raw sEMG	4.708/4.806	0.099 (−0.055, 0.251)	0.988
Level walking	Ankle	H	4.724/4.806	0.082 (−0.103, 0.255)	0.751
Level walking	Joint mean	Raw sEMG	5.516/5.666	0.150 (−0.057, 0.348)	0.901
Level walking	Joint mean	H	5.603/5.666	0.062 (−0.181, 0.293)	0.988
Stair ascent	Hip	Raw sEMG	5.901/6.949	1.049 (0.833, 1.270)	<0.001
Stair ascent	Hip	H	5.885/6.949	1.064 (0.842, 1.292)	<0.001
Stair ascent	Knee	Raw sEMG	6.579/7.974	1.395 (1.165, 1.607)	<0.001
Stair ascent	Knee	H	6.480/7.974	1.494 (1.262, 1.719)	<0.001
Stair ascent	Ankle	Raw sEMG	5.030/5.817	0.787 (0.625, 0.940)	<0.001
Stair ascent	Ankle	H	4.998/5.817	0.819 (0.655, 0.983)	<0.001
Stair ascent	Joint mean	Raw sEMG	5.837/6.913	1.077 (0.906, 1.242)	<0.001
Stair ascent	Joint mean	H	5.788/6.913	1.126 (0.948, 1.304)	<0.001
Stair descent	Hip	Raw sEMG	6.404/6.769	0.366 (0.092, 0.623)	0.011
Stair descent	Hip	H	6.497/6.769	0.273 (0.025, 0.516)	0.320
Stair descent	Knee	Raw sEMG	7.534/8.496	0.962 (0.665, 1.257)	<0.001
Stair descent	Knee	H	7.486/8.496	1.010 (0.696, 1.331)	<0.001
Stair descent	Ankle	Raw sEMG	4.893/5.354	0.461 (0.336, 0.587)	<0.001
Stair descent	Ankle	H	4.881/5.354	0.473 (0.321, 0.633)	<0.001
Stair descent	Joint mean	Raw sEMG	6.277/6.873	0.596 (0.412, 0.781)	<0.001
Stair descent	Joint mean	H	6.288/6.873	0.585 (0.391, 0.785)	<0.001
Slope ascent	Hip	Raw sEMG	6.324/7.033	0.709 (0.537, 0.882)	<0.001

Slope ascent	Hip	H	6.427/7.033	0.606 (0.406, 0.802)	<0.001
Slope ascent	Knee	Raw sEMG	6.403/7.393	0.991 (0.796, 1.182)	<0.001
Slope ascent	Knee	H	6.238/7.393	1.155 (0.949, 1.368)	<0.001
Slope ascent	Ankle	Raw sEMG	4.791/5.541	0.750 (0.612, 0.884)	<0.001
Slope ascent	Ankle	H	4.652/5.541	0.889 (0.735, 1.048)	<0.001
Slope ascent	Joint mean	Raw sEMG	5.839/6.656	0.816 (0.689, 0.947)	<0.001
Slope ascent	Joint mean	H	5.773/6.656	0.883 (0.733, 1.036)	<0.001
Slope descent	Hip	Raw sEMG	6.651/6.968	0.317 (0.063, 0.582)	0.296
Slope descent	Hip	H	6.771/6.968	0.197 (−0.080, 0.485)	1.000
Slope descent	Knee	Raw sEMG	7.218/8.062	0.845 (0.582, 1.100)	<0.001
Slope descent	Knee	H	7.176/8.062	0.887 (0.593, 1.174)	<0.001
Slope descent	Ankle	Raw sEMG	4.843/6.247	1.404 (1.172, 1.644)	<0.001
Slope descent	Ankle	H	4.920/6.247	1.327 (1.082, 1.570)	<0.001
Slope descent	Joint mean	Raw sEMG	6.237/7.092	0.855 (0.667, 1.050)	<0.001
Slope descent	Joint mean	H	6.289/7.092	0.804 (0.600, 1.018)	<0.001

Table S6. Effect of temporal circular shifting on ExtraTrees reconstruction performance.

Task	Target	Input	Aligned/shifted RMSE (°)	RMSE reduction (95% CI), °	Holm-adjusted P
Level walking	Hip	Raw sEMG	5.824/5.927	0.103 (−0.041, 0.237)	0.05
Level walking	Hip	H	6.006/6.003	−0.003 (−0.216, 0.199)	0.658
Level walking	Knee	Raw sEMG	6.015/6.156	0.142 (0.030, 0.251)	0.003
Level walking	Knee	H	6.080/6.109	0.029 (−0.103, 0.152)	0.658
Level walking	Ankle	Raw sEMG	4.708/4.794	0.086 (0.005, 0.165)	0.05
Level walking	Ankle	H	4.724/4.789	0.065 (−0.035, 0.159)	0.193
Level walking	Joint mean	Raw sEMG	5.516/5.626	0.110 (0.016, 0.203)	0.004
Level walking	Joint mean	H	5.603/5.634	0.030 (−0.093, 0.151)	0.658
Stair ascent	Hip	Raw sEMG	5.901/6.722	0.822 (0.643, 0.998)	<0.001
Stair ascent	Hip	H	5.885/6.731	0.846 (0.662, 1.022)	<0.001
Stair ascent	Knee	Raw sEMG	6.579/7.616	1.037 (0.866, 1.204)	<0.001
Stair ascent	Knee	H	6.480/7.527	1.047 (0.871, 1.216)	<0.001
Stair ascent	Ankle	Raw sEMG	5.030/5.639	0.609 (0.501, 0.712)	<0.001
Stair ascent	Ankle	H	4.998/5.587	0.589 (0.485, 0.690)	<0.001
Stair ascent	Joint mean	Raw sEMG	5.837/6.659	0.822 (0.685, 0.957)	<0.001
Stair ascent	Joint mean	H	5.788/6.615	0.828 (0.690, 0.960)	<0.001
Stair descent	Hip	Raw sEMG	6.404/6.650	0.247 (0.145, 0.347)	<0.001
Stair descent	Hip	H	6.497/6.703	0.206 (0.094, 0.315)	<0.001
Stair descent	Knee	Raw sEMG	7.534/8.033	0.499 (0.404, 0.594)	<0.001
Stair descent	Knee	H	7.486/7.987	0.501 (0.391, 0.610)	<0.001
Stair descent	Ankle	Raw sEMG	4.893/5.156	0.263 (0.203, 0.323)	<0.001
Stair descent	Ankle	H	4.881/5.156	0.275 (0.181, 0.372)	<0.001
Stair descent	Joint mean	Raw sEMG	6.277/6.613	0.336 (0.268, 0.407)	<0.001
Stair descent	Joint mean	H	6.288/6.615	0.328 (0.251, 0.405)	<0.001
Slope ascent	Hip	Raw sEMG	6.324/6.864	0.540 (0.427, 0.655)	<0.001
Slope ascent	Hip	H	6.427/6.895	0.468 (0.334, 0.599)	<0.001

Task	Target	Input	Aligned/shifted RMSE (°)	RMSE reduction (95% CI), °	Holm-adjusted P
Slope ascent	Knee	Raw sEMG	6.403/7.095	0.692 (0.576, 0.813)	<0.001
Slope ascent	Knee	H	6.238/6.918	0.680 (0.549, 0.818)	<0.001
Slope ascent	Ankle	Raw sEMG	4.791/5.417	0.626 (0.519, 0.731)	<0.001
Slope ascent	Ankle	H	4.652/5.302	0.650 (0.532, 0.765)	<0.001
Slope ascent	Joint mean	Raw sEMG	5.839/6.459	0.619 (0.527, 0.714)	<0.001
Slope ascent	Joint mean	H	5.773/6.372	0.599 (0.493, 0.707)	<0.001
Slope descent	Hip	Raw sEMG	6.651/6.877	0.226 (0.085, 0.370)	0.004
Slope descent	Hip	H	6.771/6.993	0.221 (0.068, 0.379)	0.041
Slope descent	Knee	Raw sEMG	7.218/7.475	0.258 (0.149, 0.359)	<0.001
Slope descent	Knee	H	7.176/7.413	0.237 (0.125, 0.350)	<0.001
Slope descent	Ankle	Raw sEMG	4.843/5.511	0.668 (0.528, 0.813)	<0.001
Slope descent	Ankle	H	4.920/5.481	0.561 (0.427, 0.701)	<0.001
Slope descent	Joint mean	Raw sEMG	6.237/6.621	0.384 (0.290, 0.479)	<0.001
Slope descent	Joint mean	H	6.289/6.629	0.340 (0.242, 0.441)	<0.001
